# Peripheral Inflammatory Cytokines and Lymphocyte Subset Features of Deceased COVID-19 Patients

**DOI:** 10.1155/2021/9101082

**Published:** 2021-01-22

**Authors:** Nan Jiang, Zhijun Li, Bo Yang, Mengdi Jin, Yaoyao Sun, Yang He, Yang Liu, Yueying Wang, Daoyuan Si, Piyong Ma, Jinnan Zhang, Tianji Liu, Qiong Yu

**Affiliations:** ^1^Department of Emergency, China-Japan Union Hospital of Jilin University, Changchun 130021, China; ^2^Department of Epidemiology and Biostatistics, School of Public Health, Jilin University, Changchun 130021, China; ^3^Institute of Organ Transplantation, Tongji Hospital, Tongji Medical College, Huazhong University of Science and Technology, Wuhan 430000, China; ^4^Department of Cardiology, China-Japan Union Hospital of Jilin University, Changchun 130021, China; ^5^Department of Critical Care Unit, China-Japan Union Hospital of Jilin University, Changchun 130021, China; ^6^Department of Neurosurgery, China-Japan Union Hospital of Jilin University, Changchun 130021, China

## Abstract

**Objective:**

To compare the difference of inflammatory cytokines and lymphocyte subsets between deceased patients and survivors with COVID-19.

**Methods:**

This retrospective study included 254 confirmed patients from 10 January to 11 March, 2020, at Tongji Hospital of Wuhan, China. Laboratory and immunologic features were collected and analyzed, and the main outcomes focused on inflammatory cytokines and lymphocyte subsets.

**Results:**

A trend of markedly higher levels of inflammatory cytokines as well as lower lymphocyte subset levels in deceased patients was observed compared with survivors. ROC curve analyses indicated that inflammatory cytokines and the decrease levels of T cell, Th (helper T cells) cell, Ts (suppressor T cells) cell, B cell, and NK cell along with Th/Ts ratio increase could be used to predict the death of COVID-19. Multivariate analyses showed that higher levels of IL-6, IL-8, and IL-10 remained significantly correlated with shorter survival time and that the amount of Ts cells was negatively associated with the possibility of death in COVID-19 patients. In conclusion, SARS-CoV-2 would cause lymphopenia and result in decreased lymphocyte subset cells, particularly in Ts cell counts, which further induces hyperinflammatory response and cytokine storm. IL-6, IL-8, IL-10, and Ts cell might be independent predictors for the poor outcome of COVID-19.

## 1. Introduction

SARS coronavirus 2 (SARS-CoV-2) is a member of *β*-coronavirus (*β*-CoV) lineage B belonging to CoV family, which has been confirmed for causing coronavirus disease 2019 (COVID-19) first identified and confirmed in Wuhan, China, in December 2019 [1, 2]. Genomic analysis indicated that SARS-CoV-2 shares about 80% identical genome and almost all encoded proteins with human severe acute respiratory syndrome (SRAS-CoV), while only 50% genomic identity with middle east respiratory syndrome (MERS-CoV) and more similarities to bat CoVs [3, 4]. Considering the highly contagious nature from person-to-person and global epidemic of SARS-CoV-2, WHO firstly declared it as a Public Health Emergency of International Concern at January 30th, 2020 [5], and then characterized it as a pandemic at March 11th, 2020 [6]. Currently, there are more than 50 COVID-19 vaccine candidates in clinical trials globally, and some vaccines have been approved for emergency use in United Kingdom, China, Arab nations (the United Arab Emirates, Bahrain, and Egypt), Republic of Chile, Canada and US, and so on [7].

Previous studies demonstrated that SARS-CoV-2 infection would cause severe respiratory symptoms, lymphopenia, increased levels of peripheral inflammatory cytokines, and cytokine storm which could be related with high severity and mortality of COVID-19 [8–12]. Besides, lymphocyte subsets play an important functional role in the maintenance of humoral and cytotoxic immunity [13]. Recently, several studies had investigated the association of immunological features and lymphocyte subset alteration with the disease severity in COVID-19, whose results found SARS-CoV-2 might have a main impact on T lymphocytes, especially helper T (Th) and suppressor T (Ts) cells [13–15]. Decreased Ts cells might be an independent biomarker for severe COVID-19, and decreased Ts and B cells as well as increased Th/Ts ratio might be the independent predictors of poor treatment efficacy [13]. However, there is still insufficient knowledge about inflammatory cytokines, lymphocyte subsets, and immune response associated with the death of COVID-19.

In our study, we mainly aimed to compare and elucidate the difference and clinical significance of inflammatory cytokines and lymphocyte subsets between deceased COVID-19 patients and survivors, which might help further demonstrate the immunological effect in deceased COVID-19.

## 2. Material and Methods

### 2.1. Study Design and Population

We retrospectively recruited 254 laboratory-confirmed COVID-19 patients from 10 January to 11 March, 2020, at Tongji Hospital of Wuhan, China. All patients in the study have been confirmed with no infection of HBV, HCV, and HIV. Ethical Committee of Tongji Hospital of Huazhong University of Science and Technology (No.TJ-IRB20200364) and China-Japan Union Hospital of Jilin University (No.2020032607) approved the study and waived the written informed consent for rapid emerging infections.

### 2.2. Data Collection

All cases were confirmed on admission by nucleic acid and antibody tests. Information from electronic medical records was retrospectively collected and extracted, including demographic characteristics, medical history, routine blood and urine tests, biochemical detection, and inflammatory cytokines and lymphocyte subset tests. All data were extracted with the unified data collection form by two independent researchers. The severity of patients was classified by the Novel Coronavirus Pneumonia Diagnosis and Treatment Guidance (Seventh revised trial version) [16].

### 2.3. Statistical Analysis

Categorical variables were described as percentages and compared by chi-squared or Fisher's exact test; continuous variables were described as means (SD) or medians (interquartile range (IQR)) and compared by nonparametric comparative tests or *t*-tests. Spearman rank correlation coefficient was used to analyze the correlations between paired data. Area under the receiver operating characteristic curve (AUROC) was performed to evaluate the predictive values of peripheral inflammatory cytokines and lymphocyte subsets in predicting the mortality of COVID-19. Kaplan-Meier analyses with Log-rank test were performed to assess the clinical significance for survival time between high and low levels of inflammatory cytokines. Multivariate Cox regression model was performed to determine the predictive factors for deceased COVID-19. All statistical analyses were accomplished by the SPSS 22.0 and R 3.6.1 software, and results with *P* < 0.05 were considered statistical significant.

## 3. Results

### 3.1. Demographics and Baseline Characteristic of Deceased Cases of COVID-19

254 patients were included with 51 (20.1%) deceased. The median age of all cases was 67 years (IQR, 58-73) and 70 years (IQR, 64-76) for deceased patients. Male patients were 130 (51.2%) including 32 (62.7%) deceased cases. The occurrence of any comorbidities in all patients reached 57.4% and 58.8% in deceased patients. Hypertension (39.6%) and diabetes (19.8%) were the most common comorbidities. Fever (85.1%), cough (85.1%), dyspnea (66.0%), expectoration (64.4%), myalgia or fatigue (63.4%), and chest tightness (53.9%) were most common symptoms; less common manifestations included nausea or vomiting (29.7%), sore throat (29.7%), abdominal pain (27.7%), and hemoptysis (27.5%). The median of hospitalization time was 22 days (IQR 12-29) in all patients, 9 days (IQR 5-16) in deceased patients, and 25 days (IQR 16-30) in survivors.

### 3.2. Laboratory Findings of Deceased COVID-19 Patients on Admission

Compared with normal range, levels of leucocytes and neutrophils in deceased patients were significantly elevated above upper limit of normal range (Table 1). The counts of lymphocytes were all significantly decreased below lower limit of normal range in deceased patients. Leukocytes, neutrophils, lymphocytes, and platelets were decreased in 14 (5.5%), 10 (3.9%), 151 (59.4%), and 38 (15.0%) patients. The number of patients who developed lymphopenia in deceased cases was 49 (96.1%). Counts of leucocytes, neutrophils, monocytes, and neutrophil-to-lymphocyte ratio (NLR) in deceased patients were markedly higher than survivors (all *P* < 0.001), while levels of lymphocytes and platelets were all significantly lower in deceased patients (Table 1).

Levels of Glu, ALT, AST, ALP, and GGT were all significantly elevated in deceased cases (all *P* < 0.001) (Table 1). TP, albumin, and TC levels were all markedly lower in deceased cases (all *P* < 0.001). LDH, CRP, D-dimer, NT-proBNP, PCT, and cTnI concentrations were all markedly increased in deceased cases (all *P* < 0.001) (Table 1).

In routine urine tests, the positive rates of urine protein, urine ketone body (KET), and urobilinogen (URO) were 54.6%, 16.4%, and 5.3%, respectively (Table 1). Compared with survivors, those positive rates of deceased patients were significantly higher (all *P* < 0.001). Urine specific gravity (USG) and pH levels of all patients were within the normal range. However, the level of USG in deceased cases was significantly elevated (all *P* < 0.001) while pH levels decreased (*P* < 0.05).

Blood gas analyses showed that the median arterial partial pressure of oxygen level in deceased patients was 68 mmHg, and the median of partial pressure of carbon dioxide was 31.9 mmHg. Total partial pressure of carbon dioxide, arterial partial pressure of oxygen, standard bicarbonate, base excess of extracellular fluid, total carbon dioxide, and oxygen saturation were significantly lower in deceased patients than in survivors (all *P* < 0.05) (Table 1).

### 3.3. Peripheral Inflammatory Cytokines and Lymphocyte Subset Levels with Deceased COVID-19

**Figure 1:**
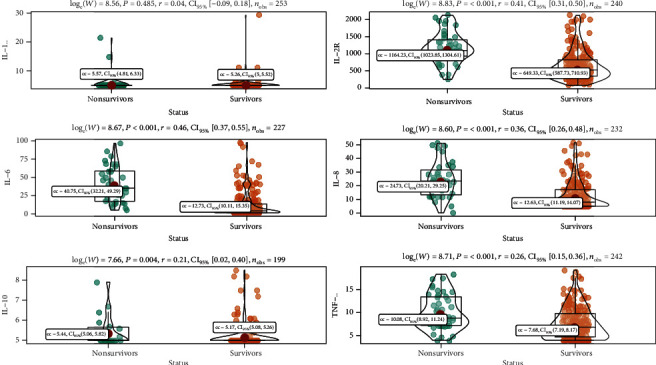
Peripheral inflammatory cytokine levels between survivors and nonsurvivors.

The level of interleukin-1*β* (IL-1*β*) was undetectable (<5 pg/mL) in almost all patients so that no statistical significance was observed between groups (Table 2). Levels of interleukin-2R (IL-2R), interleukin-6 (IL-6), interleukin-8 (IL-8), interleukin-10 (IL-10), and tumor necrosis factor *α* (TNF-*α*) on admission were significantly elevated in 120 (47.2%), 136 (53.5%), 20 (7.9%), 49 (19.3%), and 93 (36.6%) patients (Table 2). Compared with survivors, these levels were significantly higher in deceased patients (all *P* < 0.001) (Table 2, Figure 1).

**Figure 2:**
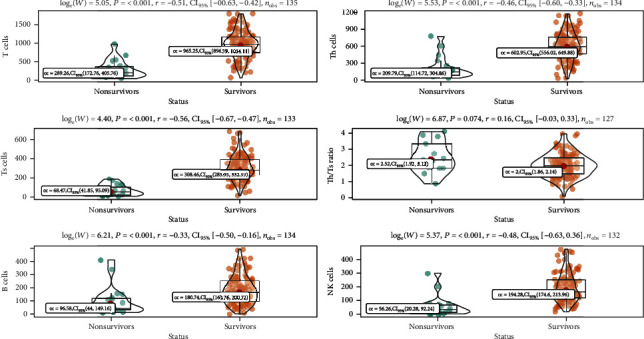
Peripheral lymphocyte subset counts between survivors and nonsurvivors.

Total counts of T cells and suppressor T (Ts) cells were both below lower limit of normal ranges, while counts of B cells, helper T (Th) cells, Ts cells, NK cells, and Th/Ts ratio were within the normal limits (Table 2). Counts of lymphocytes and T cell subsets were significantly decreased in deceased cases (all *P* < 0.001). Th/Ts ratios were significantly elevated beyond upper limit of normal range (3.32 vs. 2.00/*μ*L, *P* < 0.001). Counts of T, B, Th, Ts, and NK cells were decrease in 71/137 (51.8%), 39/137 (28.5%), 65/137 (47.4%), 85/137 (62.0%), and 64/137 (46.7%) cases. Th/Ts ratio was increased in 29 (21.2%) patients. Meanwhile, counts of T, B, Th, Ts, and NK cells were markedly lower in deceased cases, but Th/Ts ratio was higher (all *P* < 0.001) (Table 2, Figure 2).

### 3.4. Correlation Analysis between Cytokine Profiles and Lymphocyte Subsets in COVID-19

**Figure 3:**
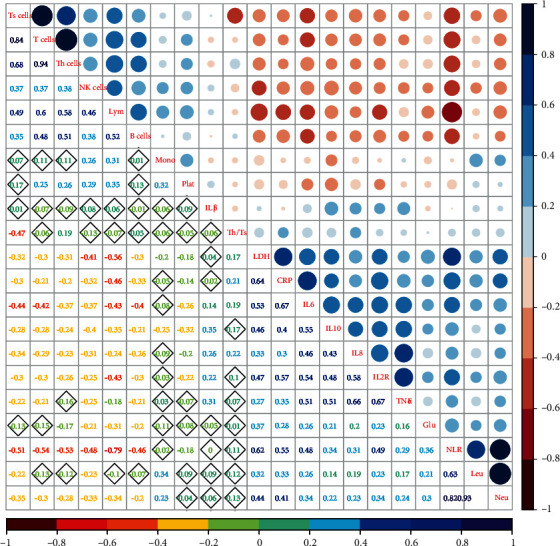
Correlation analysis of peripheral inflammatory cytokines and lymphocyte subsets in COVID-19. (Blue means positive correlation, red means negative correlation, circle size represents for strength of correlation, the number in plot means correlation coefficient, and diamond means no statistical significance.)

Total counts of T, Ts, B, and NK cells were negatively related with levels of LDH, CRP, IL-2R, IL-6, IL-8, IL-10, and TNF-*α* (all *P* < 0.05). Except for TNF-*α* (*P* = 0.064), counts of Th cells was negatively correlated with numbers of neutrophils (*P* < 0.001) and platelets (*P* = 0.002) and concentrations of CRP (*P* = 0.002), LDH (*P* < 0.001), CRP (*P* = 0.023), IL-2R (*P* = 0.002), IL-6 (*P* < 0.001), IL-8 (*P* = 0.008), and IL-10 (*P* = 0.006). Total numbers of T, Ts, and NK cells were also negatively correlated with neutrophil counts (all *P* < 0.001), and Ts, B, and NK cells with leucocytes (all *P* < 0.05). Numbers of B and NK cells were negatively correlated with glucose level (*P* < 0.05). NK cell count was also negatively correlated with numbers of monocytes (*P* = 0.002) and platelets (*P* = 0.002) (Figure 3). Details of the correlation between all the collected data are shown in *Supplementary file* see (available here).

### 3.5. Predictive Values of Potential Markers for Distinguishing Deceased COVID-19

**Figure 4:**
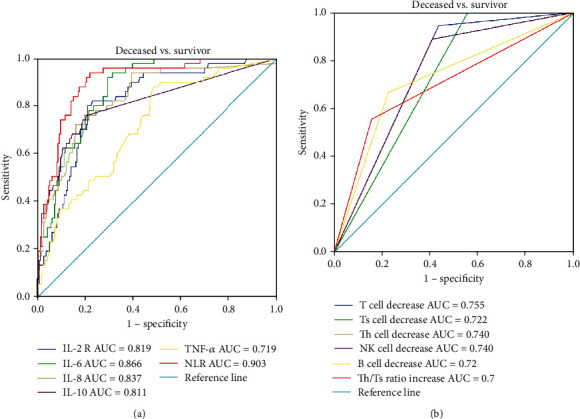
ROC curve of inflammatory cytokines (a) and lymphocyte subsets (b) in deceased COVID-19 patients on admission.

ROC curve analyses indicated that the plasma levels of inflammatory cytokines could predict the mortality of COVID-19 as all AUCs were above 0.7 (Figure 4(a)). ROC curve analyses in lymphocyte subsets indicated that the decrease of T, Th, Ts, B, and NK cell counts along with Th/Ts ratio increase could be used to predict the occurrence of death in COVID-19 patients (all *P* < 0.05) (Figure 4(b)).

**Figure 5:**
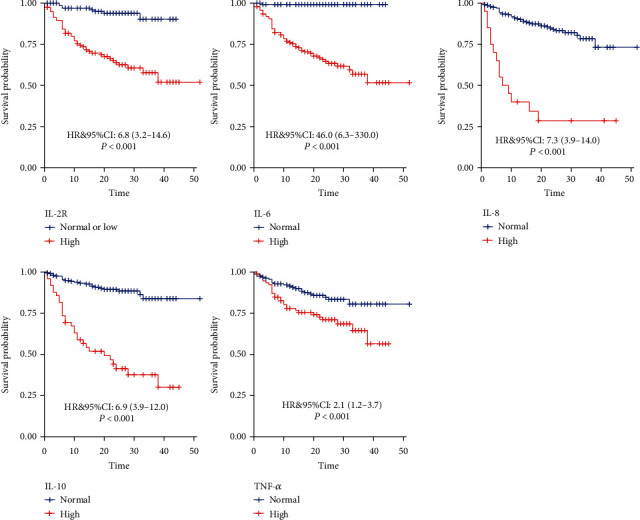
Survival according to the levels of inflammatory cytokines in COVID-19.

Kaplan-Meier survival curves showed that higher IL-2R, IL-6, IL-8, IL-10, and TNF-*α* levels were significantly correlated with reduced survival time in COVID-19 (all *P* < 0.001) (Figure 5).

**Figure 6:**
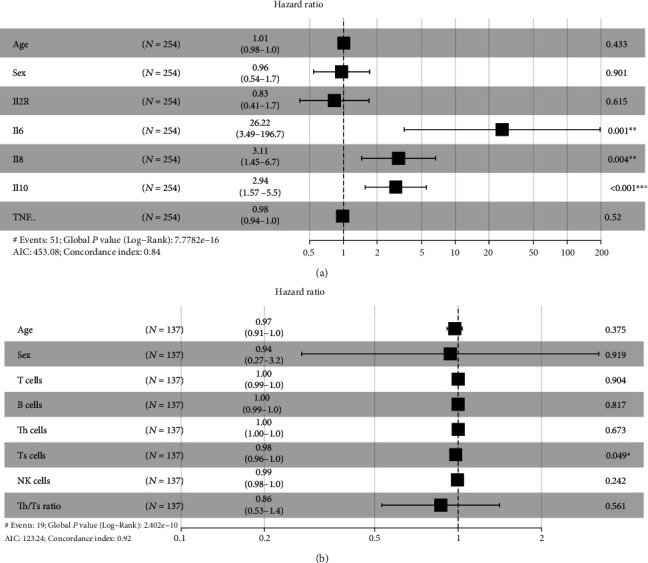
Multivariate Cox analyses of inflammatory cytokines (higher vs. normal or lower) and lymphocyte subsets (/*μ*L) with deceased COVID-19.

Multivariate Cox model analyses showed that higher levels of IL-6, IL-8, and IL-10 remained significantly correlated with shorter survival time (IL-6: HR 26.22, *P* = 0.001; IL-8: HR 3.11, *P* = 0.004; IL-10: HR 2.94, *P* < 0.001), and Ts cell count (/*μ*L) was negatively associated with COVID-19-related death (HR 0.98, *P* = 0.049) (Figure 6(b)), indicating that IL-6, IL-8, and IL-10 levels and Ts cell count could be the independent risk factors to predict the possibility of death of COVID-19.

## 4. Discussion

Previous studies suggested that several coronavirus infections could cause sustained responses of cytokine storm and dysregulation in lymphocyte subset levels, resulting in high incidence of immune disorders and mortality [13, 17]. Although a number of researches on clinical and epidemiological characteristics have been published [9, 11, 18], there is still insufficient knowledge of the potential mechanism in COVID-19. Thus, elucidating the features of peripheral inflammatory cytokines and lymphocyte subsets would be important for exploring the immune response mechanism of COVID-19.

Our laboratory findings showed that most patients presented lymphopenia, increased neutrophil count, and elevated levels of glucose, NT-proBNP, procalcitonin, cTnI, ALT, AST, LDH, CRP, and D-dimer. In deceased cases, there were significantly higher numbers of leucocytes and neutrophils and markedly higher levels of NLR, glucose, NT-proBNP, procalcitonin, cTnI, ALT, AST, LDH, CRP, and D-dimer than survivors but significantly lower numbers of lymphocytes, monocyte, and platelets. Leukocytosis, lymphopenia, and increased concentration of acute phase reactants were more commonly seen in deceased cases, which could promote death from COVID-19. Patients with uncontrolled blood glucose or diabetes had longer median length of stay and significantly higher mortality than those with well-controlled blood glucose or nondiabetes [19–21]. The increased neutrophil count, NLR, and D-dimer might indicate higher incidence of poor clinical outcomes [10, 14, 22] and myocardial injury which might be induced by inflammation was related with cardiac insufficiency, arrhythmias, and adverse endpoints [23]. Recently, one study using machine learning tools has identified 3 indicators (LDH, lymphocyte, and hs-CRP) which could predict the death of COVID-19 more than 10 days in advance with the accuracy of over 90% and found that high levels of LDH require immediate medical attention in patients [24]. Previous studies had demonstrated that acute phase reactants play key roles in the diagnosis and prediction of systemic inflammation and infection as well as the prognosis in severe patients of SARS and MERS [25–27], which were consistent with our findings. Further research on acute phase reactants would help with early diagnosis, prediction of disease process, and clinical outcomes.

Our results showed that all inflammation cytokine levels were substantially elevated, which is characterized as cytokine storm, to be significantly higher in most of the deceased cases similar with SARS and MERS results [28, 29]. Previous studies had suggested that inflammation cytokines and chemokines hold vital positions in cytokine storm [30]. Elevated concentrations of IL-6, IL-8, and IL-10 remained significantly correlated with shorter survival time on multivariate analyses, indicating that the association between baselines of IL-6, IL-8, and IL-10 and shorter survival time in COVID-19 might be the independent predictor of poor clinical outcomes and an addition to prognostic factors. Elevated concentration of IL-6 had been indicated as a stable indicator for the progression and adverse endpoint of COVID-19, and IL-6 and IL-10 were significantly related with the positivity duration of SARS-CoV-2 [31, 32], which were consistent with our findings. Previous studies showed that IL-6R antagonist tocilizumab could reverse the cytokine storm [33], and specifically blockading the signaling pathways regulated by IL-6 might be a promising method to reduce inflammation-related injuries and the mortality [34, 35]. IL-10 has a major anti-inflammatory effect regulated by JAK-STAT pathway [36], produced by antigen presentation cells (APC) including dendritic cells, macrophages, and Th cells [36, 37]. Higher IL-8 levels were found in deceased cases that might induce the hyperinflammatory response, while only S protein in all SARS proteins could induce the activation of IL-8 promoter [38, 39]. Anti-Spike-IgG (Anti-S-IgG) in SARS-CoV-2 infection would promote proinflammatory monocyte or macrophage accumulation and IL-8 production in lungs [40]. Additionally, a single center, randomised, open-label, phase 2 trial of anti-IL-8 therapy (BMS-986253) compared with Standard of Care in treating Hospitalized severe patients with COVID-19 (NCT04347226) is ongoing for improving the health condition of patients.

Hyperinflammatory cytokines may be associated with tissue injury, respiratory failure, multiple organ failure, or shock [31]. Besides, cytokines released under the innate immune responses against virus infection could also induce the release of glucocorticoids and other peptides by the neuroendocrine system, resulting in impaired immune response [31]. SARS-CoV-2 viral proteins might target several innate immune signaling proteins from pathways including the interferon (IFN) pathway, NF-*κ*B pathway, and JAK/STAT signaling pathway [15, 41] that play crucial roles in excessive inflammation and immunopathology against virus infection, causing increased inflammatory cytokines/chemokines levels. Additionally, previous studies indicated that therapies using cytokines or cytokine inhibitors have been successively successful [42], and interferon *α* of antipathogenic cytokines is an officially recommended drug for treating COVID-19 in China [43]. The dysregulation of immune response would induce the hyperinflammatory even cause mortality. However, the underling role of cytokine storms in COVID-19 pathogenesis is still unclear and needs to further research.

Direct cytopathic effects and immune evasion have been proved to play important roles in disease severity of coronavirus infection [17]. Here, we demonstrated that lymphocytes and T cell subset counts showed significant reduction in most cases of COVID-19, but B cell counts decreased only in 28.5% of them. T cell and Ts cell counts decreased beyond the lower limit of normal range, while numbers of Th, B, and NK cells remained within normal range. In deceased cases, all lymphocytes and T cell subset counts reduced markedly below the lower limit of normal range, which were significantly lower than survivors. One previous study suggested that lymphocyte subset levels in severe cases had significantly reduced, excluding only B cells [15] which was different with our results. This might due to the fact that there were only 6 out of 8 severe cases in their study and no available immunological data in moderate cases. In our study, Th/Ts ratio elevated in 21.2% patients and was markedly higher in deceased cases than survivors. The reduction in counts of all lymphocyte subsets was correlated with both severity and mortality of COVID-19 [44], but Th/Ts ratio only correlated with mortality. However, only Ts cell was found significantly associated with deceased COVID-19 on multivariate analyses, indicating that Ts cell might be the potential predictor for deceased COVID-19, which was consistent with previous studies [13, 44]. We also found that counts of lymphocyte subsets were all negatively correlated with IL-6, IL-8, and IL-10 levels. Previous studies indicated that IL-6 could suppress normal T cell activation while IL-8 would attract neutrophil granulocytes and T lymphocytes, which might partly explain the lymphopenia [38, 39]. Besides, IL-6 and IL-8 induced by SARS-CoV would impair T cell functions on prime dendritic cells as well as viral clearance of macrophages and dendritic cells, thus causing impairment of adaptive immune response [33]. As for IL-10, it could indirectly inhibit T cell activation and production of T cell-derived cytokines by the inhibitory effects on APCs as well as directly suppress production of some cytokines [45]. IL-10 could also mediate direct effects on T cells, B cells, NK cells, mast cells, and eosinophils [45]. It was reported that IL-10 signaling blockade at the priming stage could induce stronger Ts cell responses, which is beneficial for treatment of chronic viruses and cancer [37]. Our study demonstrated that the depletion of lymphocyte subsets might cause hyperinflammatory response in deceased cases, resulting in overproduction of proinflammatory cytokines and cytokine storm in SARS-CoV-2 infection, which might be part of the immunogenicity of COVID-19.

Mechanism of lymphocyte reduction in deceased COVID-19 is still unclear. Previous studies revealed that lymphopenia might be correlated with apoptosis activation and P53 signaling pathway in lymphocytes [46]. Similar with SARS-CoV and MERS-CoV [47, 48], these infections could induce apoptosis in T cells and cause significantly decrease in numbers of peripheral lymphocyte subsets, which would further induce apoptosis in multiple organs and T lymphocytes. Moreover, different exposure doses of SARS-CoV-2 could also affect lymphocyte response. High dose exposure would cause adverse endpoints and viral clearance delay, which might be related to the inefficient T and B cell responses caused by lymphopenia, subsequently inducing cytokine storm and destructive tissue inflammation [41]. It is believed that lymphocytes, especially Th and Ts cells, serve major roles in attenuating overactivated innate immune response in SARS-CoV-2 infection [15, 49]. In this process, Th cell helps promoting virus-specific antibodies production, regulating immune responses, and mediating the activation, proliferation, and deletion of immune cells (especially Ts cells), which may result in the finding that high-level Th cell count had a strong correlation with the severity of SARS-CoV infection [47]. Besides, Ts cells are critical for regulating viral clearance and immune-mediated injury, which can be strongly affected by lymphopenia; Ts and NK cells are essential for facilitating appropriate antiviral responses, which might be associated with the elevated level of NK group 2 member A (NKG2A) [49, 50]. Glucocorticoid treatment in COVID-19 might induce the decrease in lymphocytes [13, 15], and drugs on lymphocyte proliferation or preventing apoptosis might be beneficial for preventing lymphopenia or restoring lymphocyte numbers in severe cases [51].

Several limitations may exist in the study. Firstly, this is a retrospective study in which we only estimated the number of lymphocyte and T cell subsets on admission with severity and mortality in COVID-19. The percentage, temporal change, and function of lymphocyte subsets together with the dynamics of cellular immune response after SARS-CoV-2 infection might need to be elucidated by the large-sample study. Secondly, there is an impairment of immune system after SARS-CoV-2 infection that might cause coinfection with various pathogens or secondary infection, possibly influencing the effect of immune response in COVID-19.

In conclusion, SARS-CoV-2 would induce lymphopenia with decreased levels of lymphocyte subset cells, particularly Ts cells, and subsequently induce hyperinflammatory response and cytokine storm. Levels of IL-6, IL-8, IL-10, and Ts cell count might be the independent predictors of disease severity and adverse endpoints and additions to prognostic factors. Further researches on the immune pathogenesis and effect are of importance for the clinical management and development of therapeutics and vaccines of COVID-19.

## Figures and Tables

**Table 1:** Laboratory findings of deceased patients with COVID-19 on admission.

Biomarker	Normal range	Median (IQR)			*P* value
		All patients (254)	Survivors (203)	Nonsurvivors (51)	
Leucocytes, ×10^9^/L	3.5-9.5	6.20 (4.81-8.93)	5.87 (4.64-7.27)	10.66 (6.29-13.4)	<0.001
	Increased	56 (22.0%)	26 (12.8%)	30 (58.8%)	<0.001
	Decreased	14 (5.5%)	12 (5.9%)	2 (3.9%)	
Neutrophils, ×10^9^/L	1.8-6.3	4.38 (3.17-7.26)	3.96 (2.80-5.31)	9.48 (5.87-12.59)	<0.001
	Increased	75 (29.5%)	37 (18.2%)	38 (74.5%)	<0.001
	Decreased	10 (3.9%)	9 (4.4%)	1 (2.0%)	
Lymphocytes, ×10^9^/L	1.1-3.2	0.96 (0.64-1.40)	1.09 (0.74-1.51)	0.56 (0.43-0.73)	<0.001
	Decreased	151 (59.4%)	102 (50.2%)	49 (96.1%)	<0.001
NLR	1.6-5.7	4.8 (2.3-9.6)	3.7 (2.0-6.1)	15.3 (9.6-26.9)	<0.001
	Decreased	24 (9.4%)	24 (11.8%)	—	0.003
	Increased	106 (41.7%)	57 (28.1%)	49 (96.1%)	
Monocyte, ×10^9^/L	0.1-0.6	0.49 (0.36-0.63)	0.51 (0.40-0.64)	0.37 (0.28-0.59)	0.002
Platelets, mean (SD), ×10^9^/L	125-350	227.1 ± 101.2	243 ± 99	164 ± 85	<0.001
	Increased	31 (12.2%)	30 (14.8%)	1 (2.0%)	<0.001
	Decreased	38 (15.0%)	21 (10.3%)	17 (33.3%)	
ESR, mm/H	0-20 mm/H	40 (18.5-64.0)	41 (20-66)	36 (15-63)	0.299
Glu, mmol/L	4.11-6.05	6.13 (5.29-8.07)	5.83 (5.16-7.20)	8.99 (6.65-11.77)	<0.001
NT-proBNP, pg/mL	<738	244 (96.2-849.8)	192 (74-540)	1235 (601-4390)	<0.001
PCT, ng/mL	0.02-0.05	0.06 (0.03-0.16)	0.04 (0.03-0.10)	0.22 (0.12-1.01)	<0.001
cTnI, pg/mL	≤15.6	6.6 (2.5-24.2)	4.5 (2.2-11.9)	94.7 (22.0-908.7)	<0.001
Myoglobin, ng/mL	≤106	56.2 (34.1-119.7)	48.6 (31.2-89.4)	160.0 (82.6-442.9)	<0.001
CK-MB, ng/mL	≤3.4	0.8 (0.5-1.8)	0.7 (0.4-1.4)	2.8 (1.4-7.4)	<0.001
Blood biochemistry					
ALT, U/mL	≤33	24.5 (15.8-42.0)	23 (15-40)	29 (19-50)	0.023
AST, U/mL	≤32	26.0 (19.0-45.0)	24 (18-37)	47 (30-79)	<0.001
TP, g/L	64-83	68.6 (64.4-72.8)	69.3 (65.4-73.6)	63.8 (56.9-70.5)	<0.001
Albumin, g/L	35-53	33.2 (30.5-37.5)	34.6 (31.4-38.1)	29.7 (25.3-32.5)	<0.001
Globulin, U/mL	25-35	34.1 (30.6-38.1)	34.0 (31.4-38.1)	34.3 (31.3-38.6)	0.946
TBIL, *μ*mol/L	≤2	10.5 (7.2-14.6)	9.5 (7.0-13.9)	14.0 (9.6-19.2)	<0.001
DBIL, *μ*mol/L	≤8	4.4 (3.1-6.6)	4.1 (3.0-5.7)	6.2 (4.4-10.2)	<0.001
IBIL, *μ*mol/L	≤12.9	5.4 (4.1-8.2)	5.2 (3.9-7.9)	6.5 (4.5-9.2)	0.046
ALP, U/L	35-105	71 (55-93)	67 (55-87)	84 (58-122)	0.003
GGT, U/L	6-42	30 (19-54.5)	29 (18-52)	40 (20-81)	0.046
TC, mmol/L	<5.18	3.77 (3.12-4.41)	3.9 (3.3-4.5)	3.2 (2.7-3.9)	<0.001
LDH, U/L	135-214	292.5 (227.8-453.0)	265 (214-351)	532 (469-760)	<0.001
Creatinine, *μ*mol/L	45-85	74 (60-92.5)	71 (59-88)	88 (69-160)	<0.001
CRP, mg/L	<1	28.1 (4.8-92.5)	13.8 (2.5-61)	102.9 (62.5-187.1)	<0.001
	Increased	216 (85.0%)	165 (86.8%)	51 (100.0%)	0.003
Coagulation function					
PT, s	11.5-14.5	14.0 (13.4-14.9)	13.8 (13.2-14.3)	15.5 (14.6-18.1)	<0.001
INR	0.8-1.2	1.08 (1.01-1.17)	1.05 (0.99-1.11)	1.22 (1.14-1.47)	<0.001
Fibrinogen, g/L	2-4	5.00 (3.42-6.08)	5.08 (3.71-6.13)	3.92 (2.61-5.82)	0.004
APTT, s	29-42	39.1 (36.1-43.4)	39 (36.2-43.0)	39.1 (35.7-45.1)	0.984
D-dimer, U/mL FEU	<0.5	1.46 (0.57-4.05)	1.18 (0.48-2.18)	15.01 (1.77-21.00)	<0.001
Urine routine					
Urine protein, n/*N* (%)	—	123/225 (54.7%)	84/185 (45.4%)	39/40 (97.5%)	<0.001
USG, n, median (IQR)	1.01-1.025	225, 1.016 (1.012-1.022)	185, 1.015 (1.011-1.020)	40, 1.021 (1.018-1.028)	<0.001
pH, n, median (IQR)	4.5-8.0	225, 6.5 (6.0-6.5)	185, 6.5 (6.0-6.75)	40, 6.0 (6.0-6.5)	0.039
KET, n/*N* (%)	—	37/225 (16.4%)	18/185 (9.7%)	19/40 (47.5%)	<0.001
URO, n/*N* (%)	—	12/225 (5.3%)	6/185 (3.2%)	6/40 (15.0%)	0.009
Arterial blood gas					
pH, *N*, median (IQR)	7.35-7.45	77, 7.42 (7.39-7.46)	58, 7.41 (7.39-7.45)	19, 7.42 (7.38-7.48)	0.382
PaCO2, mmHg	35-45	76, 38.8 (34.4-43.2)	57, 40.7 (37.4-43.7)	19, 31.9 (25.3-37.4)	<0.001
PaO2, mmHg	80-100	76, 117 (78.5-174.8)	57, 134 (87.2-187)	19, 68 (51.7-121)	<0.001
SB, mmol/L	21-25	75, 25.3 (23.5-27.2)	56, 25.4 (23.9-27.3)	19, 22.2 (19.0-25.3)	0.001
BE, mmol/L	-3-3	76, 0.8 (-1.3-3.2)	57, 1.3 (-0.5-3.3)	19, -3.9 (-8.1-0.2)	0.001
TCO2, mmol/L	24-32	75, 22.5 (20.2-24.2)	56, 23.0 (21.4-24.4)	19, 17.8 (16.0-22.5)	0.001
SaO2, *N*, median (IQR)	91.9-99%	76, 98.6 (94.0-99.6)	57, 99.1 (97.2-99.6)	19, 89.8 (85.1-97.5)	<0.001

NLR: neutrophil-to-lymphocyte ratio; ESR: erythrocyte sedimentation rate; Glu: glucose; ALT: alanine aminotransferase; AST: aspartate aminotransferase; ALP: alkaline phosphatase; TP: total protein; TBIL: total bilirubin; DBIL: direct bilirubin; IBIL: indirect bilirubin; ALP: alkaline phosphatase; GGT: gamma-glutamyl transpeptidase; TC: total cholesterol; LDH: lactate dehydrogenase; CRP: C-reactive protein; NT-proBNP: N-terminal of the prohormone brain natriuretic peptide; PCT: procalcitonin; cTnI: cardiac troponin I; CK-MB: creatine kinase-M; PT: prothrombin time; INR: international normalized ratio; APTT: activated partial thromboplastin time; USG: urine specific gravity; KET: urine ketone body; URO: urobilinogen; PaCO2: partial pressure of carbon dioxide; PaO2: arterial partial pressure of oxygen; SB: standard bicarbonate; BE: base excess of extracellular fluid; TCO2: total carbon dioxide; SaO2: oxygen saturation; *N*: number of patients; IQR: interquartile range.

**Table 2:** Inflammatory cytokines and lymphocyte subsets in deceased patients with COVID-19 on admission.

Biomarker	Normal range	*N*, median (IQR)			*P* value
		All patients (254)	Survivors (203)	Nonsurvivors (51)	
Inflammatory cytokines					
Interleukin-1*β*, pg/mL	<5	254, 5 (5-5)	203, 5 (5-5)	51, 5 (5-5)	0.235
Interleukin-2R, U/mL	223-710	254, 644 (387-1105)	203, 541 (350.0-868.0)	51, 1220 (949-1861)	<0.001
	Decreased	19 (7.5%)	19 (9.4%)	—	<0.001
	Increased	120 (47.2)	112 (55.2)	8 (15.7%)	
Interleukin-6, pg/mL	<7	254, 8.15 (2.00-40.47)	203, 4.9 (1.5-19.3)	51, 58.5 (26.88-137.48)	<0.001
	Increased	136 (53.5%)	86 (42.4%)	50 (98.0%)	<0.001
Interleukin-8, pg/mL	<62	254, 11.6 (5.1-24.0)	203, 8.3 (5.0-18.0)	51, 29.8 (20.6-70.7)	<0.001
	Increased	20 (7.9%)	6 (3.0%)	14 (27.5%)	<0.001
Interleukin-10, pg/mL	<9.1	254, 5 (5-6.4)	203, 5 (5-5)	51, 9.8 (5.2-18.3)	<0.001
	Increased	49 (19.3%)	20 (9.9%)	29 (56.9%)	<0.001
TNF-*α*, pg/mL	<8.1	254, 7.5 (5.2-11.2)	203, 6.9 (4.7-10.3)	51, 10.4 (7.5-14.8)	<0.001
	Increased	93 (36.6%)	65 (32.0%)	28 (54.9%)	0.003
Lymphocyte subsets					
T cells+B cells+NK cells, n/*μ*L	—	137, 1309.0 (862.5-1612.5)	118, 1394 (1052.5-1654.2)	19, 333 (171-748)	<0.001
T cells, n/*μ*L	955-2860	137, 939 (534.5-1155.5)	118, 976 (753.8-1188.2)	19, 201 (122-392)	<0.001
	Decreased	71/137 (51.8%)	53/118 (44.9%)	18/19 (94.7%)	<0.001
B cells, n/*μ*L	90-560/*μ*L	137, 158.0 (81.5-250.5)	118, 167.5 (100-254.5)	19, 41 (30-126)	<0.001
	Decreased	39/137 (28.5%)	27/118 (22.9%)	12/19 (63.2%)	<0.001
Th cells, n/*μ*L	550-1440/*μ*L	137, 562.0 (330.0-761.5)	118, 606.5 (466-796)	19, 147 (84-249)	<0.001
	Decreased	65/137 (47.4%)	48/118 (40.7%)	17/19 (89.5%)	<0.001
Ts cells, n/*μ*L	320-1250/*μ*L	137, 270.0 (174.5-387.0)	118, 294.5 (233.8-406.5)	19, 54 (20-124)	<0.001
	Decreased	85/137 (62.0%)	66/118 (55.9%)	19/19 (100.0%)	<0.001
NK cells, n/*μ*L	150-1100/*μ*L	137, 153.0 (95.0-253.5)	118, 172.5 (122-263.8)	19, 35 (10-70)	<0.001
	Decreased	64/137 (46.7%)	47/118 (39.8%)	17/19 (89.5%)	<0.001
Th/Ts ratio	0.71-2.78	137, 2.06 (1.52-2.70)	118, 2.00 (1.48-2.54)	19, 3.32 (1.87-5.44)	<0.001
	Increased	29/137 (21.2%)	19/118 (16.1%)	10/19 (52.6%)	<0.001

*N*: number of patients; IQR: interquartile range; T cells: CD3 + CD19- T cells; B cells: CD3-CD19+ T cells; Th, helper T cells: CD3 + CD4+ T cells; Ts, suppressor T cells: CD3 + CD8+ T cells; NK cells: CD3-/CD16 + CD56+ T cells.

## Data Availability

The datasets analyzed in the study are available from our corresponding author by request.

## Supplementary Materials

Supplementary Materials Correlation coefficient and *P* value between cytokine profiles and lymphocyte subsets in COVID-19.
