# Supplementary material for: Peripheral Inflammatory Cytokines and Lymphocyte Subset Features of Deceased COVID-19 Patients
Source: Biomed Res Int. 2021 Jan 22;2021:9101082. doi: 10.1155/2021/9101082 (PMC7841449; doi:10.1155/2021/9101082)
Supplement: Supplementary Materials — Correlation coefficient and P value between cytokine profiles and lymphocyte subsets in COVID-19. [file 9101082.f1.pdf]

Supplementary file: Correlation coefficient and p value between cytokines profiles and lymphocyte subsets in COVID-19

	lower	r	upper	p.value
Leu-Neu	0.906445933	0.925562654	0.940894196	2.53E-116
Leu-Lym	-0.220049245	-0.104050293	0.014850268	0.086168408
Leu-Mono	0.226299881	0.335990395	0.437265935	1.25E-08
Leu-Plat	-0.033926664	0.085133348	0.201810811	0.160698167
Leu-Glu	0.090060299	0.208298449	0.320746451	0.000644104
Leu-LDH	0.212781343	0.323728618	0.426434457	4.98E-08
Leu-CRP	0.210319437	0.326796311	0.434125447	1.57E-07
Leu-IL1β	-0.032454538	0.090993498	0.211706951	0.148164244
Leu-IL2R	0.108507063	0.228539345	0.342003452	0.000239643
Leu-IL6	0.146559277	0.26463738	0.375280061	1.85E-05
Leu-IL8	0.07056599	0.191983054	0.307794376	0.002117391
Leu-IL10	0.014932449	0.137763794	0.256498994	0.028146282
Leu-TNFα	0.047262197	0.169361529	0.286474295	0.006821467
Leu-T cells	-0.293551148	-0.132351744	0.036166549	0.12313828
Leu-B cells	-0.234647249	-0.069673699	0.099200988	0.418502881
Leu-Th cells	-0.283890733	-0.121983216	0.046687425	0.155606674
Leu-Ts cells	-0.372128176	-0.218019657	-0.052213365	0.010487184
Leu-NK cells	-0.383310126	-0.230406811	-0.065211268	0.006755207
Leu-Th/Tr	-0.049402783	0.119301189	0.28138651	0.164968267
Leu-NLR	0.554145677	0.631329773	0.69775072	9.15E-32
Neu-Lym	-0.437530876	-0.336280998	-0.2266107	1.22E-08
Neu-Mono	0.113355898	0.228991417	0.33850618	0.000135062
Neu-Plat	-0.07799087	0.041106854	0.159047809	0.498808169
Neu-Glu	0.183706917	0.297617365	0.40364045	8.05E-07
Neu-LDH	0.342374206	0.443438921	0.534359516	1.75E-14
Neu-CRP	0.303404552	0.41281258	0.511477418	1.53E-11
Neu-IL1β	-0.06816637	0.055382854	0.177259068	0.379412865
Neu-IL2R	0.222905376	0.336750761	0.441534238	3.76E-08
Neu-IL6	0.226867925	0.34022864	0.44449378	2.50E-08
Neu-IL8	0.111813952	0.231709582	0.34495603	0.000194963
Neu-IL10	0.10235614	0.22263582	0.33649927	0.000349276
Neu-TNFα	0.117860078	0.237499283	0.350342449	0.00013277
Neu-T cells	-0.441889258	-0.296119696	-0.135114517	0.00044246
Neu-B cells	-0.357698955	-0.202108277	-0.035599495	0.017863399
Neu-Th cells	-0.426741621	-0.278994067	-0.116741085	0.000961941
Neu-Ts cells	-0.493271341	-0.354917946	-0.199051017	2.09E-05
Neu-NK cells	-0.475368168	-0.334305742	-0.176485487	6.54E-05
Neu-Th/Tr	-0.035687344	0.132823133	0.293989563	0.121797174
Neu-NLR	0.774913369	0.818346324	0.854087387	3.85E-67
Lym-Mono	0.203683082	0.31478841	0.417888707	1.08E-07
Lym-Plat	0.246111431	0.354471726	0.454080303	1.67E-09
Lym-Glu	-0.411823092	-0.306536038	-0.193172655	3.59E-07
Lym-LDH	-0.633141367	-0.555926497	-0.467756276	2.20E-23
Lym-CRP	-0.554131828	-0.461009721	-0.356491677	2.39E-14
Lym-IL1β	-0.059046538	0.064506828	0.186113673	0.305804224
Lym-IL2R	-0.525560419	-0.430312055	-0.324409794	7.15E-13
Lym-IL6	-0.527291847	-0.432461496	-0.326990184	4.81E-13
Lym-IL8	-0.354635023	-0.24211899	-0.122690762	9.70E-05
Lym-IL10	-0.452264371	-0.348584384	-0.23560871	1.14E-08
Lym-TNFα	-0.298440669	-0.182043192	-0.060309991	0.003598749
Lym-T cells	0.475503724	0.595711605	0.694081379	1.61E-14
Lym-B cells	0.388714405	0.522374509	0.634501163	5.87E-11
Lym-Th cells	0.460027492	0.582779374	0.683671991	7.94E-14
Lym-Ts cells	0.351941218	0.490693061	0.608343786	1.15E-09
Lym-NK cells	0.320982285	0.463733294	0.585881684	1.15E-08
Lym-Th/Tr	-0.230719952	-0.065540577	0.103310517	0.446703368
Lym-NLR	-0.828126156	-0.786757312	-0.736864485	1.01E-58
Mono-Plat	0.204147289	0.315224695	0.418288391	1.03E-07
Mono-Glu	-0.228888372	-0.111463996	0.009157637	0.070052053
Mono-LDH	-0.310755467	-0.198967781	-0.081750849	0.000990289
Mono-CRP	-0.172815327	-0.048796678	0.076745079	0.446118541
Mono-IL1β	-0.180343191	-0.058558492	0.064994514	0.352649004
Mono-IL2R	-0.155041969	-0.032579136	0.0908698	0.605294274
Mono-IL6	-0.200805391	-0.079934614	0.043333453	0.203295377
Mono-IL8	-0.205992273	-0.085064489	0.038422347	0.176545335

Mono-IL10	-0.363785982	-0.251984395	-0.13302569	4.87E-05
Mono-TNFα	-0.092931313	0.030502132	0.15301222	0.628500268
Mono-T cells	-0.057681642	0.111108724	0.273723469	0.196154807
Mono-B cells	-0.153162789	0.014936381	0.182195458	0.86246777
Mono-Th cells	-0.06095876	0.107859453	0.270678449	0.209639075
Mono-Ts cells	-0.096811413	0.07207433	0.236925862	0.402610572
Mono-NK cells	0.094972956	0.258569831	0.40856732	0.002282262
Mono-Th/Tr	-0.225883983	-0.060458936	0.108355263	0.482796339
Mono-NLR	-0.140819465	-0.022478125	0.096496501	0.711574609
Plat-Glu	-0.198761032	-0.08018281	0.040709154	0.19318753
Plat-LDH	-0.292721149	-0.179838697	-0.06201246	0.002967401
Plat-CRP	-0.260669245	-0.140165667	-0.015361602	0.027943556
Plat-IL1β	-0.031015796	0.092421578	0.213082175	0.141876789
Plat-IL2R	-0.336926976	-0.223094258	-0.10283346	0.000339325
Plat-IL6	-0.370343782	-0.259297706	-0.140944626	2.76E-05
Plat-IL8	-0.31150423	-0.19593198	-0.074647648	0.001702746
Plat-IL10	-0.430712339	-0.324849365	-0.210168167	1.18E-07
Plat-TNFα	-0.189560101	-0.068063607	0.055485779	0.279849013
Plat-T cells	0.086275169	0.250367913	0.401235299	0.003169766
Plat-B cells	-0.040679318	0.127908835	0.289415692	0.136344605
Plat-Th cells	0.092154108	0.255914269	0.406195526	0.002541269
Plat-Ts cells	-0.000819574	0.166918857	0.325521413	0.051231731
Plat-NK cells	0.12403656	0.285806459	0.432777134	0.000710406
Plat-Th/Tr	-0.214590896	-0.048625449	0.120069233	0.572573551
Plat-NLR	-0.293338811	-0.180922185	-0.063570465	0.002696182
Glu-LDH	0.260027065	0.368964956	0.468628475	5.72E-10
Glu-CRP	0.161398825	0.281768575	0.393877808	8.54E-06
Glu-IL1β	-0.172211655	-0.049446828	0.074830507	0.435419114
Glu-IL2R	0.113937183	0.234448857	0.34816118	0.000178106
Glu-IL6	0.143169882	0.262105582	0.373578544	2.51E-05
Glu-IL8	0.073372412	0.19541626	0.31169362	0.001867291
Glu-IL10	0.092955184	0.214307767	0.329386546	0.000630541
Glu-TNFα	0.039779777	0.162797107	0.280953011	0.009778215
Glu-T cells	-0.314851622	-0.154718819	0.013978113	0.072096612
Glu-B cells	-0.359343594	-0.203308723	-0.036215255	0.01759983
Glu-Th cells	-0.329934142	-0.171104174	-0.00285348	0.046404491
Glu-Ts cells	-0.292004322	-0.130064628	0.03912486	0.131242784
Glu-NK cells	-0.361281861	-0.20544313	-0.038439598	0.01642214
Glu-Th/Tr	-0.160791225	0.007751462	0.175854884	0.928633889
Glu-NLR	0.254429	0.363774898	0.463937136	1.03E-09
LDH-CRP	0.560992456	0.641236854	0.709542926	8.95E-30
LDH-IL1β	-0.085554731	0.03792923	0.160265579	0.547355601
LDH-IL2R	0.363538042	0.465780803	0.556935978	4.41E-15
LDH-IL6	0.432966245	0.527740062	0.610973836	1.09E-19
LDH-IL8	0.220818218	0.334803051	0.439765276	4.55E-08
LDH-IL10	0.353918502	0.457091286	0.549273242	1.62E-14
LDH-TNFα	0.155644727	0.273489904	0.383659558	9.79E-06
LDH-T cells	-0.441461786	-0.295635116	-0.134593102	0.000452594
LDH-B cells	-0.444817469	-0.299441121	-0.138690813	0.000378442
LDH-Th cells	-0.456522867	-0.312753662	-0.153067099	0.000198486
LDH-Ts cells	-0.460713657	-0.317533616	-0.158245543	0.000156255
LDH-NK cells	-0.542988753	-0.412872591	-0.263395992	5.34E-07
LDH-Th/Tr	0.002304768	0.169954561	0.328311844	0.047087825
LDH-NLR	0.542103846	0.621136943	0.689277481	2.62E-30
CRP-IL1β	-0.14542521	-0.019415557	0.107213939	0.764275024
CRP-IL2R	0.481398559	0.572913195	0.652071612	2.01E-22
CRP-IL6	0.598081846	0.673398183	0.736924217	2.48E-33
CRP-IL8	0.185596633	0.304814218	0.415188323	1.42E-06
CRP-IL10	0.290747732	0.402332192	0.503119402	8.60E-11
CRP-TNFα	0.231822851	0.347994918	0.454380058	2.88E-08
CRP-T cells	-0.37111163	-0.210506911	-0.037673903	0.017524269
CRP-B cells	-0.480777078	-0.334586905	-0.170298984	0.000120681
CRP-Th cells	-0.362229646	-0.200678997	-0.027423104	0.023682471
CRP-Ts cells	-0.452771691	-0.302411073	-0.135326009	0.00054911
CRP-NK cells	-0.468120687	-0.320003441	-0.154396285	0.000244911
CRP-Th/Tr	0.036474704	0.209358986	0.370075705	0.018163426
CRP-NLR	0.455269977	0.549077682	0.63082888	8.98E-21

IL1β-IL2R	0.097645631	0.218108802	0.332273203	0.000463198
IL1β-IL6	0.019604233	0.142345306	0.260859464	0.023269103
IL1β-IL8	0.138679772	0.257371166	0.368773553	3.30E-05
IL1β-IL10	0.23826096	0.351050516	0.454496762	8.86E-09
IL1β-IL1β	0.194737409	0.310382352	0.417516408	4.48E-07
IL1β-T cells	-0.232919948	-0.065281518	0.1061246	0.455333142
IL1β-B cells	-0.17655065	-0.006519803	0.163888879	0.940627601
IL1β-Th cells	-0.255133695	-0.088761885	0.082714696	0.309631119
IL1β-Ts cells	-0.164512763	0.005878656	0.175929394	0.946457039
IL1β-NK cells	-0.089577396	0.081898218	0.248658395	0.348678144
IL1β-Th/Tr	-0.226240684	-0.058257519	0.113090792	0.505358307
IL1β-NLR	-0.125541369	-0.00249538	0.120626219	0.968432989
IL2R-IL6	0.442484092	0.536357051	0.618603114	2.54E-20
IL2R-IL8	0.489033183	0.577364746	0.653974786	5.59E-24
IL2R-IL10	0.376313081	0.477290341	0.56706168	7.41E-16
IL2R-TNFα	0.596645954	0.670491049	0.733076896	1.59E-34
IL2R-T cells	-0.447256428	-0.299859444	-0.136605562	0.000453951
IL2R-B cells	-0.449953798	-0.30292966	-0.139917984	0.000393759
IL2R-Th cells	-0.415687845	-0.264152827	-0.098348358	0.002123689
IL2R-Ts cells	-0.446944631	-0.299504745	-0.136223117	0.000461427
IL2R-NK cells	-0.405296346	-0.252489377	-0.08595706	0.003367615
IL2R-Th/Tr	-0.07566209	0.095798559	0.261756757	0.27268546
IL2R-NLR	0.388667093	0.488387704	0.576799138	1.25E-16
IL6-IL8	0.361958524	0.464355345	0.55568002	5.47E-15
IL6-IL10	0.453609834	0.546198814	0.627122756	3.73E-21
IL6-TNFα	0.416599225	0.513360238	0.598619959	1.76E-18
IL6-T cells	-0.548638761	-0.417393619	-0.266071638	5.79E-07
IL6-B cells	-0.53512233	-0.401465937	-0.248201286	1.67E-06
IL6-Th cells	-0.504656015	-0.365858669	-0.208624812	1.49E-05
IL6-Ts cells	-0.5691189	-0.441681939	-0.293524078	1.03E-07
IL6-NK cells	-0.506003038	-0.367424453	-0.210354379	1.36E-05
IL6-Th/Tr	0.024669947	0.194081694	0.352657423	0.025191024
IL6-NLR	0.383072279	0.483177668	0.57206541	2.54E-16
IL8-IL10	0.319854695	0.426161615	0.52187194	1.25E-12
IL8-TNFα	0.578826895	0.655229826	0.720228876	1.54E-32
IL8-T cells	-0.439754181	-0.291336221	-0.127429048	0.000668331
IL8-B cells	-0.414917158	-0.263286282	-0.097425964	0.002199254
IL8-Th cells	-0.385715168	-0.230631418	-0.062872998	0.007567751
IL8-Ts cells	-0.481352404	-0.338894299	-0.17899344	6.60E-05
IL8-NK cells	-0.454941072	-0.308614352	-0.146060781	0.000301334
IL8-Th/Tr	0.055404164	0.22352285	0.379316797	0.009702172
IL8-NLR	0.191272501	0.307126467	0.414540345	5.98E-07
IL10-TNFα	0.41840713	0.514970956	0.600023063	1.32E-18
IL10-T cells	-0.432824748	-0.283484643	-0.119000417	0.000944536
IL10-B cells	-0.366014622	-0.208797034	-0.03999166	0.015870439
IL10-Th cells	-0.390445695	-0.235897683	-0.068418305	0.006266243
IL10-Ts cells	-0.430709148	-0.281091489	-0.116436084	0.001047508
IL10-NK cells	-0.5325219	-0.398410852	-0.24478546	2.04E-06
IL10-Th/Tr	-0.003501049	0.166825166	0.327744628	0.054954865
IL10-NLR	0.229387347	0.342793452	0.447017193	2.06E-08
TNFα-T cells	-0.369954065	-0.21315067	-0.044539965	0.013764654
TNFα-B cells	-0.368775733	-0.211847796	-0.043178101	0.014367649
TNFα-Th cells	-0.32220633	-0.16079907	0.009693042	0.064461915
TNFα-Ts cells	-0.37543781	-0.219221363	-0.050893793	0.011237126
TNFα-NK cells	-0.400082291	-0.246653788	-0.079776583	0.004209016
TNFα-Th/Tr	-0.099570862	0.071874169	0.239174679	0.411001146
TNFα-NLR	0.169107663	0.286234375	0.395388964	3.54E-06
T cells-B cells	0.338390257	0.478925167	0.598562239	3.22E-09
T cells-Th cells	0.91342187	0.937514785	0.955060603	9.28E-64
T cells-Ts cells	0.787134576	0.8434962	0.885887507	2.95E-38
T cells-NK cells	0.218862306	0.372890261	0.508786509	7.21E-06
T cells-Th/Tr	-0.222730178	-0.057149528	0.111635975	0.507118283
T cells-NLR	-0.651930427	-0.543657852	-0.4136598	6.65E-12
B cells-Th cells	0.377718061	0.512939272	0.626737844	1.47E-10
B cells-Ts cells	0.194294605	0.350585828	0.48951829	2.67E-05
B cells-NK cells	0.230179043	0.383104923	0.517565468	3.83E-06
B cells-Th/Tr	-0.122629535	0.046032702	0.212110612	0.593240915

B cells-NLR	-0.57944334	-0.456047265	-0.312211657	2.14E-08
Th cells-Ts cells	0.581297654	0.6824767	0.762872359	4.17E-20
Th cells-NK cell	0.230957212	0.383805938	0.518166918	3.66E-06
Th cells-Th/Tr	0.026408644	0.19326812	0.349650032	0.023648236
Th cells-NLR	-0.640003659	-0.529078698	-0.396551045	3.00E-11
Ts cells-NK cells	0.220321274	0.374209247	0.509921699	6.65E-06
Ts cells-Th/Tr	-0.5927433	-0.471944908	-0.330379798	5.82E-09
Ts cells-NLR	-0.623271439	-0.508735198	-0.372830655	2.19E-10
NK cells-Th/Tr	-0.288835471	-0.127285994	0.041311402	0.138279258
NK cells-NLR	-0.59586394	-0.475686471	-0.334671034	4.25E-09
Th/Tr-NLR	-0.061368291	0.107453149	0.270297456	0.211370792
